# Collective Housing of Mice of Different Age Groups before Maturity Affects Mouse Behavior

**DOI:** 10.1155/2020/6856935

**Published:** 2020-11-12

**Authors:** Hiroshi Ueno, Shunsuke Suemitsu, Shinji Murakami, Naoya Kitamura, Kenta Wani, Yu Takahashi, Yosuke Matsumoto, Motoi Okamoto, Takeshi Ishihara

**Affiliations:** ^1^Department of Medical Technology, Kawasaki University of Medical Welfare, Okayama 701-0193, Japan; ^2^Department of Psychiatry, Kawasaki Medical School, Kurashiki 701-0192, Japan; ^3^Department of Neuropsychiatry, Graduate School of Medicine, Dentistry and Pharmaceutical Sciences, Okayama University, Okayama 700-8558, Japan; ^4^Department of Medical Technology, Graduate School of Health Sciences, Okayama University, Okayama 700-8558, Japan

## Abstract

**Background:**

Although population housing is recommended by many animal management and ethical guidelines, the effect of collective housing of mice of different age groups on mouse behavior has not been clarified. Since the development of the central nervous system continues to occur before sexual maturation, the stress of social ranking formation among male individuals in mixed housing conditions can affect postmaturation behavior. To assess these effects, sexually immature mice of different ages were housed in the same cage and a series of behavioral tests were performed after maturation.

**Results:**

The findings for three groups of mice—junior mice housed with older mice, senior mice housed with younger mice, and mice housed with other mice of the same age—were compared. Junior mice showed higher body weight and activity as well as lower grip strength and anxiety-like behaviors than other mice. In contrast, senior mice showed lower body temperature and increased aggression, antinociceptive effect, and home-cage activity in the dark period in comparison with other mice.

**Conclusions:**

Thus, combined housing of immature mice of different age groups affects mouse behavior after maturation. Appropriate prematuration housing conditions are crucial to eliminate the uncontrollable bias caused by age-related social stratification.

## 1. Background

Most animals used in research and testing are kept in small cages. However, many animal care and ethical guidelines recommend raising mice in a population to reduce the stress caused by social isolation [1]. Male mice are aggressive to allogeneic male mice [2]. However, such behavior is natural for territorial animals [3]. Social ranking is formed among male individuals raised in the same cage [4]. Such established social hierarchies are stable, and inferior animals are frequently attacked by dominant animals [5].

The formation of a social hierarchy is a basic survival strategy in many animal species and helps maintain a safe social environment without wasteful conflict within the group [6]. Furthermore, this hierarchy deeply influences access to resources essential for survival, health, and reproductive success [7]. In 1963, Tinbergen showed that social behavior is influenced by genetic, physiological, and environmental factors [8]. Dominance hierarchies may be encoded by neural mechanisms [9]. However, many of the effects of social superiority and inferiority on animal health are unknown. Mice are the most widely used social animals in neuroscience research, but the influence of social hierarchies in the cage on the behavioral development of mice has not been elucidated.

The establishment of social relationships, an important determinant of social behavior in mammals, begins shortly after birth, first with the mother, followed by other members of the family and the community [10]. Puberty is an impressionable period in terms of social conditions [11]. In humans, puberty is characterized by the continued development of social capacity and behavior along with neuroanatomical maturation of the brain areas involved in social cognition [12]. Social disruption in this period may impair normal maturation of social skills and may contribute to the development of psychiatric disorders such as autism [13].

Considering the differences in behaviors such as aggression, locomotion, depression-like behavior, novel object recognition, fear conditioning, stereotypy, and manageability between group-housed male mice and isolated male mice, population housing is recommended by animal care and ethical guidelines [14]. A controlled laboratory environment is critical for animal welfare [15], and it is a well-studied topic in the field of animal welfare science [16]. However, no study has examined the effect of collective housing of mice of different ages on mouse behavior in detail. The development of the prefrontal cortex related to emotion and cognitive function normally precedes sexual maturity (adolescence); during this time, animals are more immature and are therefore more susceptible to stress than mature animals [17]. Since these species live under various ecological and social conditions after weaning, we conducted experiments using mice before maturation.

In this study, we aimed to characterize the differences in postmaturation behavior of male mice of different ages that were housed together prior to maturation. We conducted various behavioral tests to evaluate the higher brain functions in these mice. The comparisons were performed across three groups: (1) senior mice housed with younger mice, (2) junior mice housed with older mice, and (3) mice housed with those of the same age before sexual maturity. The present housing context manipulation is purely experimental. The findings of this study are of relevance for laboratory animal management. Moreover, we expect these findings to demonstrate that a mouse model may be used to elucidate the biological and environmental factors underlying social hierarchy.

## 2. Results

### 2.1. Effects of Group Housing of Mice of Different Ages on Changes in Body Weight and Temperature

**Figure 1:**
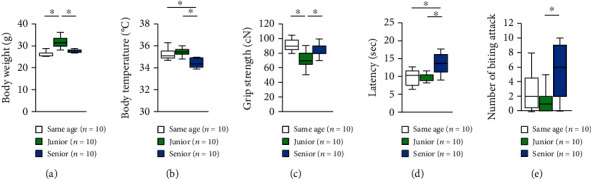
Effect of group housing condition on normal physical characteristics and aggressive behaviors. (a) Body weight. (b) Body temperature. (c) Grip strength. (d) Hot plate test. (e) Number of biting attacks on the cotton bud. All data are presented as box plots. ^∗^Significant difference in comparison between groups (*p* < 0.05). The *p* values were calculated by using two-way ANOVA.

We compared the general health of the junior, senior, and same-age groups. Body weight was significantly higher in junior mice (Figure 1(a), two-way ANOVA: *F*_2,28_ = 33.780, *p* < 0.001; post hoc Tukey test: same age vs. junior, *p* < 0.001; junior vs. senior, *p* < 0.001; same age vs. senior, *p* = 0.241). Body weight was also significantly higher in senior mice than in same-age mice. Senior mice showed a significantly lower body temperature than both same-age and junior mice (Figure 1(b), two-way ANOVA: *F*_2,28_ = 9.024, *p* = 0.001; post hoc Tukey test: same age vs. junior, *p* = 0.373; junior vs. senior, *p* = 0.001; same age vs. senior, *p* = 0.019).

### 2.2. Effects of Group Housing on Neuromuscular Strength and Aggressive Behaviors

We compared the neuromuscular strength of mice among groups. Grip strength was significantly lower in junior mice than in same-age or senior mice (Figure 1(c), two-way ANOVA: *F*_2,28_ = 6.214, *p* = 0.006; post hoc Tukey test: same age vs. junior, *p* = 0.007; junior vs. senior, *p* = 0.038; same age vs. senior, *p* = 0.845). To assess nociception, mice were placed on a hot plate. Senior mice showed a significantly higher pain threshold than either the same-age or junior mice (Figure 1(d), two-way ANOVA: *F*_2,28_ = 8.542, *p* = 0.001; post hoc Tukey test: same age vs. junior, *p* = 0.981; junior vs. senior, *p* = 0.004; same age vs. senior, *p* = 0.002). We also evaluated the aggressive behaviors of the mice. The number of biting attacks was significantly higher in senior mice than in junior mice (Figure 1(e), two-way ANOVA: *F*_2,28_ = 4.549, *p* = 0.019; post hoc Tukey test: same age vs. junior, *p* = 0.635; junior vs. senior, *p* = 0.016; same age vs. senior, *p* = 0.109).

### 2.3. Effect of Group Housing on Performance in the Elevated Plus Maze Test

**Figure 2:**
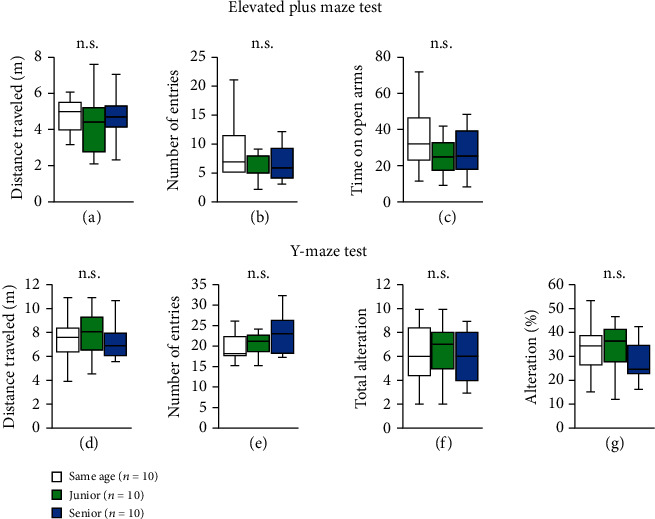
Effect of group housing condition on performance on the elevated plus maze and Y-maze tests. (a–c) Elevated plus maze test. Graphs showing the total distance traveled (a), the number of entries into the open arm (b), and the time spent in the open arms (c) in the elevated plus maze test. (d–g) Y-maze test. Graphs showing the total distance traveled (d), the total number of arm entries (e), the total number of alternations (f), and the percentage of alternations (g). All data are presented as box plots. ^∗^Significant difference in comparisons between groups (*p* < 0.05). The *p* values were calculated using two-way ANOVA.

In the elevated plus maze test, we examined the effect of group housing with mice of different ages on anxiety-like behaviors in senior and junior mice. No differences were observed in the total distance traveled (Figure 2(a), two-way ANOVA: *F*_2,28_ = 0.558, *p* = 0.578; post hoc Tukey test: same age vs. junior, *p* = 0.549; junior vs. senior, *p* = 0.886; same age vs. senior, *p* = 0.857), the total number of entries into the open arms (Figure 2(b), two-way ANOVA: *F*_2,28_ = 1.785, *p* = 0.186; post hoc Tukey test: same age vs. junior, *p* = 0.161; junior vs. senior, *p* = 0.657; same age vs. senior, *p* = 0.637), or the time spent in the open arms (Figure 2(c), two-way ANOVA: *F*_2,28_ = 0.982, *p* = 0.387; post hoc Tukey test: same age vs. junior; *p* = 0.354; junior vs. senior, *p* = 0.754; same age vs. senior, *p* = 0.816) among the three groups.

### 2.4. Effect of Group Housing on Performance in the Y-Maze Test

We examined the effect of group housing with mice of different ages on short-term spatial working memory by monitoring the spontaneous alternation behavior in the Y-maze test. There were no significant intergroup differences in the total distance traveled (Figure 2(d), two-way ANOVA: *F*_2,28_ = 0.464, *p* = 0.634; post hoc Tukey test: same age vs. junior, *p* = 0.658; junior vs. senior, *p* = 0.727; same age vs. senior, *p* = 0.997), number of arm entries (Figure 2(e), two-way ANOVA: *F*_2,28_ = 0.755, *p* = 0.479; post hoc Tukey test: same age vs. junior, *p* = 0.944; junior vs. senior, *p* = 0.651; same age vs. senior, *p* = 0.465), number of alternations (Figure 2(f), two-way ANOVA: *F*_2,28_ = 0.027, *p* = 0.974; post hoc Tukey test: same age vs. junior, *p* = 0.983; junior vs. senior, *p* = 0.975; same age vs. senior, *p* = 0.999), or alternation percentage (Figure 2(g), two-way ANOVA: *F*_2,28_ = 0.223, *p* = 0.802; post hoc Tukey test: same age vs. junior, *p* = 0.999; junior vs. senior, *p* = 0.837; same age vs. senior, *p* = 0.819).

### 2.5. Effect of Group Housing on Performance in the Open-Field Test

**Figure 3:**
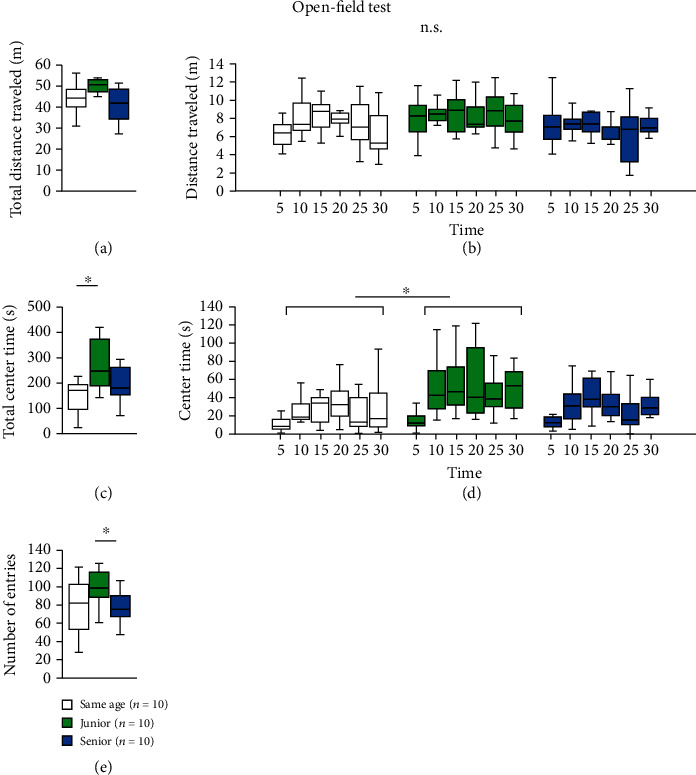
Effect of group housing condition on performance in the open-field test. Graphs showing the total distance traveled (a), the distance traveled in each 5 min period (b), the total time spent in the central area (c), the time spent in the central area in each 5 min period (d), and the number of entries into the central area (e). All data are presented as box plots. ^∗^Significant difference in comparisons between groups (*p* < 0.05). The *p* values were calculated using two-way ANOVA in (a), (c), and (e), and two-way repeated measures ANOVA in (b) and (d).

In the open-field test, there were no significant intergroup differences in the total distance traveled (Figure 3(a), two-way ANOVA: *F*_2,28_ = 2.693, *p* = 0.085; post hoc Tukey test: same age vs. junior, *p* = 0.269; junior vs. senior, *p* = 0.081; same age vs. senior, *p* = 0.742). We observed no significant difference in the distance traveled in each 5 min period among the three groups (Figure 3(b), two-way repeated measures ANOVA: group × time: *F*_5,140_ = 1.722, *p* = 0.133; Fisher's LSD test: same age vs. junior, *p* = 0.373; junior vs. senior, *p* = 0.099; same age vs. senior, *p* = 1.0). The total time spent in the central area was significantly higher in junior mice than in same-age mice (Figure 3(c), two-way ANOVA: *F*_2,28_ = 4.588, *p* = 0.019; post hoc Tukey test: same age vs. junior, *p* = 0.017; junior vs. senior, *p* = 0.124; same age vs. senior, *p* = 0.727). We observed that the time spent in the central area in each 5 min period was significantly higher in junior mice than in same-age mice (Figure 3(d), two-way repeated measures ANOVA: group × time: *F*_5,140_ = 7.814, *p* < 0.001; Fisher's LSD test: same age vs. junior, *p* = 0.019; junior vs. senior, *p* = 0.157; same age vs. senior, *p* = 1.0). There were no significant intergroup differences in the number of total entries into the central area (Figure 3(e), two-way ANOVA: *F*_2,28_ = 2.995, *p* = 0.066; post hoc Tukey test: same age vs. junior, *p* = 0.129; junior vs. senior, *p* = 0.092; same age vs. senior, *p* = 0.958).

### 2.6. Effect of Group Housing on Performance in the Social Interaction Test

**Figure 4:**
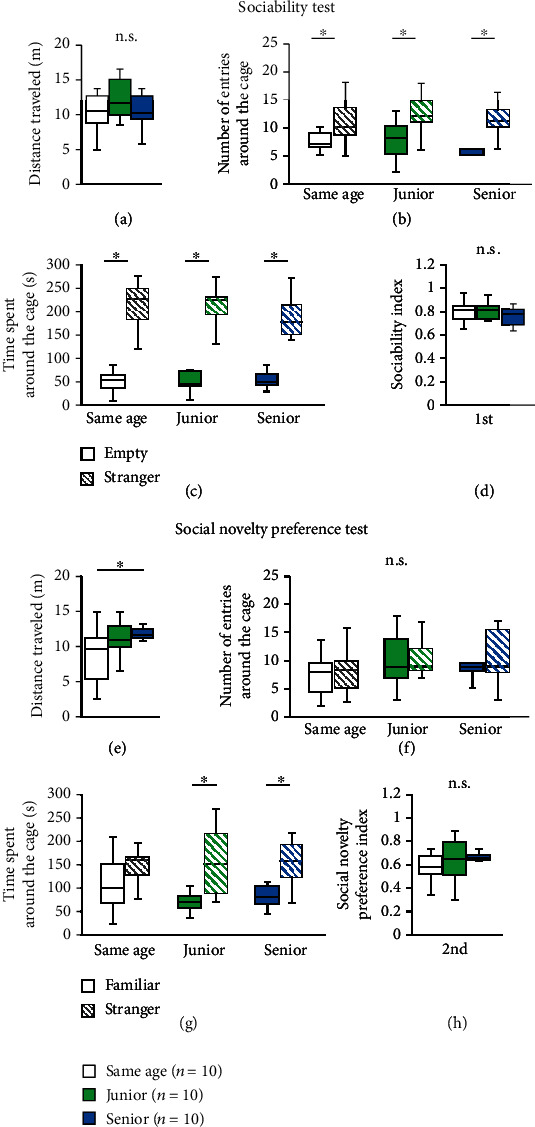
Effect of housing condition on performance in the social interaction test. The sociability test: graphs showing the total distance traveled (a), the number of entries around the cage (b), the time spent around the cage (c), and the sociability index defined as the (timespentaroundthestrangercage)/(timespentaroundthestrangercage + timespentaroundtheemptycage) (d). The social novelty preference test: graphs showing the total distance traveled (e), the number of entries around the cage (f), the time spent around the cage (g), and the social novelty preference index defined as the (timespentaroundthestrangercage)/(timespentaroundthestrangercage + timespentaroundthefamiliarcage) (h). All data are presented as box plots. ^∗^Significant difference in comparisons between groups (*p* < 0.05). The *p* values were calculated using two-way ANOVA in (a), (d), (e), and (h), and two-way repeated measures ANOVA in (b), (c), (f), and (g).

In the sociability test, the three groups showed no significant difference in the distance traveled (Figure 4(a), two-way ANOVA: *F*_2,28_ = 0.792, *p* = 0.463; post hoc Tukey test: same age vs. junior, *p* = 0.432; junior vs. senior, *p* = 0.779; same age vs. senior, *p* = 0.865). Mice of all three groups showed more entries around the cage containing a stranger mouse than around an empty cage (Figure 4(b), two-way repeated measures ANOVA: group × area: *F*_2,56_ = 0.215, *p* = 0.807; Fisher's LSD test: same age, *p* = 0.013; junior, *p* = 0.001; senior, *p* = 0.011) and spent significantly more time around the cage containing the stranger mouse than around the empty cage (Figure 4(c), two-way repeated measures ANOVA: group × area: *F*_2,56_ = 0.500, *p* = 0.609; Fisher's LSD test: same age, *p* < 0.001; junior, *p* < 0.001; senior, *p* < 0.001). There were no significant differences in the sociability index (Figure 4(d), two-way ANOVA: *F*_2,28_ = 0.238, *p* = 0.790; post hoc Tukey test: same age vs. junior, *p* = 0.791; junior vs. senior, *p* = 0.993; same age vs. senior, *p* = 0.870).

In the social novelty preference test, the total distance traveled was significantly higher in senior mice than in same-age mice (Figure 4(e), two-way ANOVA: *F*_2,28_ = 3.423, *p* = 0.047; post hoc Tukey test: same age vs. junior, *p* = 0.117; junior vs. senior, *p* = 0.897; same age vs. senior, *p* = 0.049). There were no significant differences in the number of entries around the cage containing the stranger mouse and those around the cage containing the familiar mouse in any of the three groups (Figure 4(f), two-way repeated measures ANOVA: group × area: *F*_2,56_ = 0.047, *p* = 0.953; Fisher's LSD test: same age, *p* = 0.785; junior, *p* = 0.843; senior, *p* = 0.546). Same-age mice spent a similar duration of time around both cages (Figure 4(g)). Both junior and senior mice spent a significantly longer duration of time in the area containing the stranger mouse than in the area containing the familiar mouse (Figure 4(g), two-way repeated measures ANOVA: group × area: *F*_2,56_ = 0.579, *p* = 0.563; Fisher's LSD test: same age, *p* = 0.088; junior, *p* = 0.002; senior, *p* = 0.007). There were no significant differences in the social novelty preference index (Figure 4(h), two-way ANOVA: *F*_2,28_ = 0.325, *p* = 0.725; post hoc Tukey test: same age vs. junior, *p* = 0.741; junior vs. senior, *p* = 0.998; same age vs. senior, *p* = 0.798).

### 2.7. Effect of Group Housing on Home-Cage Activity

**Figure 5:**
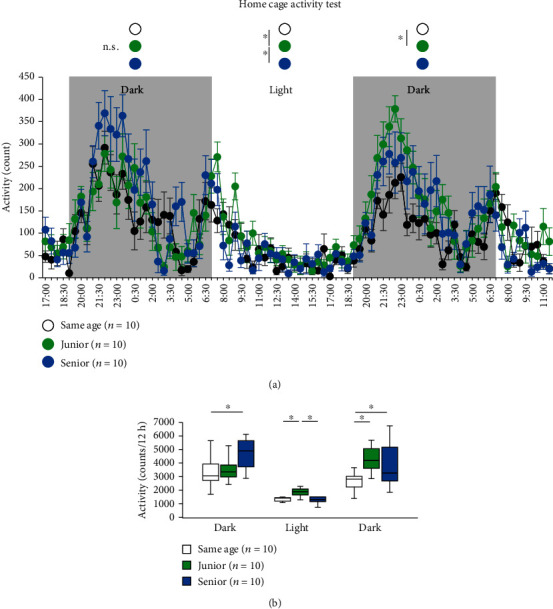
Effect of group housing condition on performance on the home-cage activity test. Graphs showing the spontaneous locomotor activity in each 30 min period (a) and total locomotor activity in the first dark phase, first light phase, and second dark phase (b). Data are presented as the mean ± SEM in (a) and box plots in (b). ^∗^Significant difference in comparisons between groups (*p* < 0.05). The *p* values were calculated using two-way repeated measures ANOVA in (a) and two-way ANOVA in (b).

We assessed home-cage activity in mice. There were no significant intergroup differences in locomotor activity during the first dark phase (Figure 5(a), first dark phase: two-way repeated measures ANOVA: *F*_23,644_ = 12.405, *p* < 0.001; Fisher's LSD test: same age vs. junior, *p* = 1.0; junior vs. senior, *p* = 0.271; same age vs. senior, *p* = 0.166; first light phase, two-way repeated measures ANOVA: *F*_23,644_ = 9.960, *p* < 0.001; Fisher's LSD test: same age vs. junior, *p* = 0.019; junior vs. senior, *p* = 1.0; same age vs. senior, *p* = 0.011; second dark phase, two-way repeated measures ANOVA: *F*_23,644_ = 10.638, *p* < 0.001; Fisher's LSD test: same age vs. junior, *p* = 0.009; junior vs. senior, *p* = 1.0; same age vs. senior, *p* = 0.079). During the first light phase, we observed a significant increase in the locomotor activity in the junior mice in comparison with the senior mice and the same-age mice (Figure 5(a)). During the second dark phase, we observed a significant increase in the locomotor activity in the junior mice compared with the same-age mice (Figure 5(a)). In the total activity counts, senior mice showed more activity in the dark phase (Figure 5(b), first dark phase, two-way ANOVA: *F*_2,29_ = 4.346, *p* = 0.023; post hoc Tukey test: same age vs. junior, *p* = 0.947; junior vs. senior, *p* = 0.052; same age vs. senior, *p* = 0.028; first light phase, two-way ANOVA: *F*_2,29_ = 5.958, *p* = 0.007; post hoc Tukey test: same age vs. junior, *p* = 0.016; junior vs. senior, *p* = 0.017; same age vs. senior, *p* = 0.992; second dark phase, two-way ANOVA: *F*_2,29_ = 6.133, *p* = 0.006; post hoc Tukey test: same age vs. junior, *p* = 0.006; junior vs. senior, *p* = 0.787; same age vs. senior, *p* = 0.046). Junior mice showed a gradual increase in activity (Figure 5(b)).

### 2.8. Effect of Group Housing on Depressive-like Behaviors in Mice

**Figure 6:**
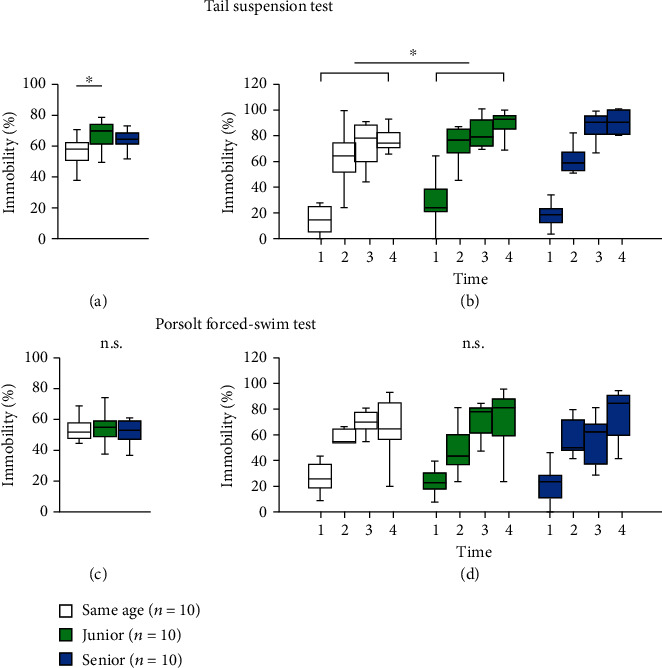
Effect of group housing condition on depressive-like behavior. Graphs showing the proportion of total time spent immobile (a) and the proportion of time spent immobile in each 1 min period (b) in the tail-suspension test. Graphs showing the proportion of total time spent immobile (c) and the proportion of time spent immobile in each 1 min period (d) in the Porsolt forced-swim test. All data are presented as box plots. ^∗^Significant difference in comparison with controls (*p* < 0.05). The *p* values were calculated using the two-way ANOVA in (a) and (c), and two-way repeated measures ANOVA in (b) and (d).

We evaluated depressive-like behaviors in the three groups. In the tail-suspension test, the total immobile time was significantly higher in junior mice than in same-age mice (Figure 6(a), two-way ANOVA: *F*_2,28_ = 4.033, *p* = 0.030; post hoc Tukey test: same age vs. junior, *p* = 0.024; junior vs. senior, *p* = 0.557; same age vs. senior, *p* = 0.270). The immobility time in each 1 min period was significantly higher in junior mice than in same-age mice (Figure 6(b), two-way repeated measures ANOVA: group × time: *F*_6,78_ = 1.086, *p* = 0.378; Fisher's LSD test: same age vs. junior, *p* = 0.027; junior vs. senior, *p* = 0.919; same age vs. senior, *p* = 0.376).

However, in the Porsolt forced-swim test, we found no significant differences in the total immobility time (Figure 6(c), two-way ANOVA: *F*_2,28_ = 0.243, *p* = 0.786; post hoc Tukey test: same age vs. junior, *p* = 0.969; junior vs. senior, *p* = 0.889; same age vs. senior, *p* = 0.771) and the immobility time in each 1 min period among the three groups (Figure 6(d), two-way repeated measures ANOVA: group × time: *F*_6,84_ = 0.804, *p* = 0.570; Fisher's LSD test: same age vs. junior, *p* = 1.0; junior vs. senior, *p* = 1.0; same age vs. senior, *p* = 1.0).

## 3. Discussion

To our knowledge, this is the first study to show that simultaneous housing of mice of different age groups in the home cage affects animal behavior postmaturation.

An important feature of living in a social group is that animals must demonstrate superior and subordinate behavior quickly and flexibly according to the relative social status of their social partners. Rats and mice show highly adaptive social behavior [18]. At high population densities, rats and mice become socially tolerant and adapt to dictatorial social systems wherein one male is socially dominant and the other male is subordinate [19]. However, changes to mouse behavior as a result of the formation of these social relationships had not been previously investigated. A recent study revealed that mouse phenotypes differ according to certain tests depending on the dominance hierarchy [20]. This study did not report findings regarding the effect of dominance hierarchy experienced during adolescence on the behavioral phenotype of mice. In the present study, we demonstrated that prematuration mice living with older mice exhibit altered behavior after maturation.

In this study, we did not investigate the hierarchical relationship between junior and senior mice in the same cage. No body injury was observed in the junior and senior mice. Moreover, frequent conflicts between the mice were not observed. A subsequent study should focus on clarifying conflicts within the cage and the hierarchical relationships between groups.

Junior mice gained significantly more weight compared to other mice. In rats, weight loss is a physical disadvantage that induces competition dependency [21]. However, one study also reported that there is no significant relationship between body weight and competitive rank in mice [22]. The weight gain observed in junior mice may be attributed to an increase in appetite due to the chronic stress of social hierarchy [23]. In addition, senior mice also showed increased body weight compared to the same-age mice. Further research is needed to investigate why the dietary intake of junior and senior mice increased.

Senior mice showed greater aggressiveness than junior mice. Aggression plays a major role in determining housing systems and social organizations [24]. Some studies have shown an association between aggressive behavior and dominance ranking [25]. This result shows that senior mice may have exerted dominance over junior mice in the same cage by means of aggression.

Body temperature of mice increases with stress [26]. In this study, senior mice had significantly lower body temperature. This result indicates that senior mice experienced significantly lower loaded stress than the other mice. Junior mice also showed lower grip strength compared to the other mice. Growth of the mouse body is affected by reduced secretion of growth hormones due to developmental stress [27]. Thus, the decrease in the grip strength of the junior mouse may be a result of acceptance of stress and social status.

Junior mice showed no change in the antinociceptive effect in comparison with the same-age housed mice. Social defeat stress does not change the thresholds in the hot plate test in mice [28]. Increased susceptibility to pain increases depression-like behavior [29]. Thus, junior mice may have been more chronically stressed. In contrast, senior mice had a significantly higher threshold in the hot plate test than the other mice. Physical traits such as height and physique impact social ranking among animals. This result suggests that senior mice maintained social superiority.

Senior mice showed increased activity in the dark period. Dominant individuals exhibit higher locomotor activity and exploratory activity [30]. Thus, the differences in the activity levels of the mouse groups within a home cage may be attributed to differences in social hierarchy.

In the open-field test, the anxiety-like behavior in junior mice was lower than that in the same-age mice. Anxiety-like behavior is known to be reduced in mice exposed to stress in early childhood [31]. In our study, the three groups showed no difference in anxiety-like behaviors in the elevated plus maze test and the Y-maze test. The open-field test is used to study anxiety-like behavior in a wide area, and the light-dark transition test is a behavioral experiment that measures anxiety in a bright place. There are several types of anxiety-like behavior, such as anxiety about heights, anxiety about bright places, and anxiety about large objects. The differences in present results are presumed to be due to the anxiety caused by tall places and the light phase [32]. Ultimately, these results show that housing with other age groups also changes the anxiety-like behavior of mice.

Social behavior changes during the developmental stage, when littermates are housed in a cage, and decreases with development [33]. In the same-age mice, sociality to stranger mice was decreased, whereas junior and senior mice had higher sociality and concern for stranger mice. This study suggests that collective housing of different age groups may also affect social behavior.

Individuals with a low social rank among four male mice were reported to show significantly greater anxiety-like behavior and depression-like behavior [4]. In addition, repeated exposure to social defeat stress triggers a series of depressed and uneasy behaviors, including anhedonia, anxiety, and social avoidance, in adult rodents [34]. Consistent with previous studies, junior mice in the present study also showed increased depression-like behaviors in the tail-suspension test compared to the same-age mice. Junior mice also had higher body weight and lower grip strength than the other mice. It is possible that the physical and neuromuscular differences affected the results of depression-like behavior. Further studies are needed to clarify the physical characteristics of junior mice.

Housing conditions such as population housing and isolation housing of mice greatly affect mouse behavior [35]. In addition, the effects of mixing and feeding animals with different genotypes and phenotypes are a growing area of research. For example, impaired memory function in transgenic mice can be improved by housing them with wild-type animals [36], and social deficits of BTBR mouse strains are alleviated by housing them with C57BL/6 mice [37]. On the other hand, simultaneous housing of the C57BL/6 and DBA/2 lines may cause stress to C57BL/6 mice and is associated with anxiogenicity [38]. Mixing of mouse strains with different or even opposite phenotypes has been shown to affect the social environment as well as behavior and physiology. However, the impact of living with individuals of different ages on mouse behavior has not been clarified. A group of domestic mice develops a strict social hierarchy as there is a need for suppressing other individuals sharing the cage. An advantage hierarchy based on combat and tracking has been identified in this context, and the hostile relationships of these mice were organized [39].

In animals with a social hierarchy, the prefrontal cortex regulates the recognition of social status [40]. The prefrontal cortex, the amygdala, and the hypothalamus are very important in regulating social flexibility in animals given the opportunity to form a social hierarchy [41]. Injuries to the prefrontal cortex of rats have been reported to increase subordinate behavior and is associated with a decrease in social status [42]. Several brain regions (particularly the prefrontal cortex mediating emotion and cognitive function) are in the developmental stage through maturity [43]. The development of the prefrontal cortex occurs at puberty, and this structure is the last to mature in the cortex [44]. Therefore, environmental factors affecting mice during this period have both histologically and functionally affected their cerebral development. Indeed, social adversity, including battle and social isolation, during puberty significantly increases depression and anxiety-like symptoms in adolescence and adulthood [45]. Dysfunction of the prefrontal cortex causes neuropsychological diseases such as schizophrenia [46]. Social status may mediate the strong inverse correlation between chronic stress and mortality [47]. Based on these findings, the present study shows the necessity to reconsider housing conditions of immature mice to ensure sound development of their brain.

Collective housing of laboratory rodents is an essential requirement by law. As recommended, we agree that mice, which are social animals, should be raised in group housing. However, our results indicate the need for immature mice to be housed after accounting for differences in age. In any case, the housing conditions need to be unified among the groups. This study raises concerns about the handling of experimental results using mice. Researchers sometimes group mice by weight. The present findings show that these operations may confound experimental results. Therefore, it is desirable to establish animal housing conditions to minimize the effects of differences in age.

## 4. Conclusion

This study shows that the behavior of mice after maturation changes significantly depending on the social hierarchy formed between mice of different ages under population housing. The reproducibility of results obtained using animal studies may be influenced by the housing conditions of the animals before maturity, specifically by the presence of animals of different ages in the cages. Appropriate housing conditions before mouse maturation are important to eliminate uncontrollable bias caused by age-related social hierarchy.

## 5. Methods

### 5.1. Animals

All animal experiments were performed in accordance with the U.S. National Institute of Health (NIH) Guide for the Care and Use of Laboratory Animals (NIH Publication No. 80-23, revised in 1996) and approved by the Committee for Animal Experiments at Kawasaki Medical School Advanced Research Center. All efforts were made to minimize the number of animals used and their suffering. Animals were purchased from Charles River Laboratories (Kanagawa, Japan) and housed in cages (five animals per cage) with food and water provided *ad libitum* under a 12 h light/dark cycle at 23°C−26°C. At the end of the experiments, mice were euthanized by cervical dislocation under anesthesia induced by carbon dioxide.

### 5.2. Group Housing

**Figure 7:**
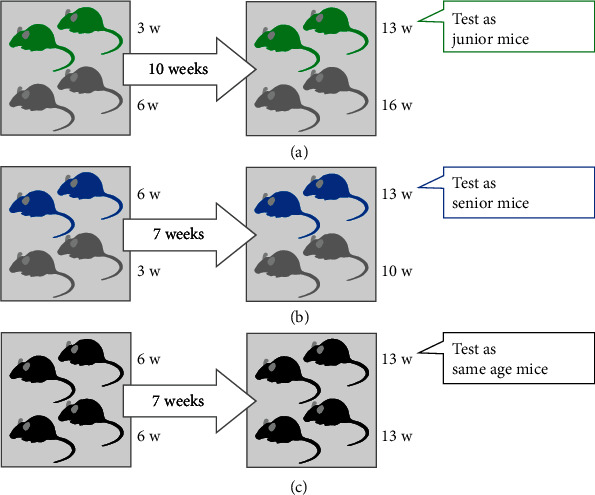
Schematic illustration of the experimental design. (a) Junior mice group: two 3-week-old mice and two 6-week-old mice were housed together in each cage. We housed the 3-week-old mice until they were 13 weeks old. (b) Senior mice group: two 6-week-old mice and two 3-week-old mice were housed together in each cage. We housed the 6-week-old mice until they were 13 weeks old. (c) Same-age mice group: four 6-week-old mice were housed together in each cage. We housed the 6-week-old mice until they were 13 weeks old.

We used male C57BL/6N mice. The animals were randomly assigned to either the junior, senior, or same-age groups (Figure 7). (1) For the junior mice group, two 3-week-old mice and two 6-week-old mice were housed together in each cage. We housed the 3-week-old mice until they were 13 weeks old (Figure 7(a)). (2) For the senior mice group, two 6-week-old mice and two 3-week-old mice were housed together in each cage. We housed 6-week-old mice until they were 13 weeks old (Figure 7(b)). (3) For the same-age mice group, four 6-week-old mice were housed together in each cage. We housed 6-week-old mice until they were 13 weeks old (Figure 7(c)). The bedding in each cage was changed every 7 days. Only cages with mice with no visible bruises were used for experiments.

Senior mice (6-week-old mice) were weaned at 3 weeks and reared with mice of the same age up to 6 weeks.

### 5.3. Behavioral Tests

All behavioral tests were performed during the light phase of the circadian cycle (09:00–16:00). Each behavioral test was separated from the next by at least 1 day. The mice were tested in a random order. To prevent any bias due to olfactory cues, the apparatus was cleaned with 70% ethanol and water with superoxidized hypochlorous acid after testing. Behavioral tests were performed in the order described below. Behavioral tests were conducted between 13 and 14 weeks of age. The cage conditions were not changed during the testing phase.

### 5.4. General Health and Neurological Screening

Physical characteristics, including body weight and rectal temperature, were recorded. Neuromuscular strength was examined using the grip strength test according to a previous study [48]. A grip strength meter was used to assess forelimb grip strength. Mice were lifted and held by the tail so that their forepaws could grasp a wire grid; they then were pulled backward gently until they released the grid. The peak force applied by the mouse forelimbs was recorded in Newtons (cN).

### 5.5. Elevated Plus Maze Test

The elevated plus maze test is an assay that is widely used in rodents to assess anxiety-like behavior [49]. The apparatus consisted of two open arms (8 × 25cm) and two closed arms of the same size with 30 cm high transparent walls. The arms were constructed of white plastic plates and were elevated to a height of 40 cm above the floor. Arms of the same type were located opposite to each other. Each mouse was placed in the central square of the maze, facing one of the closed arms, and was allowed to move freely between the two arms for 10 min. The number of arm entries, distance traveled (m), and time spent in the open arms were video recorded and analyzed using video tracking software (ANY-MAZE, Stoelting Co., Wood Dale, IL, USA).

### 5.6. Open-Field Test

The open-field test is an assay that is widely used in rodents to assess anxiety-like behavior [50]. In the open-field test, each mouse was placed in the center of the apparatus, which consisted of a square area surrounded by high walls (40 × 40 × 40cm). The total distance traveled (m) and the time spent in the central area (s) were recorded. The central area was defined as the 20 × 20cm area located in the center of the field. The test chamber was illuminated at 100 lux. Data were collected over a 30 min period. Data analysis was performed automatically using the ANY-MAZE software.

### 5.7. Y-Maze Test

Spatial working memory was measured using a Y-maze apparatus (arm length: 40 cm; arm bottom width: 3 cm; arm upper width: 10 cm; and height of wall: 12 cm). The mice were placed at the center of the Y-maze field. Visual cues were placed around the maze in the testing room and were constant throughout the testing sessions. Mice were examined with no prior learning. The total distance traveled (m), the number of entries, and alterations were recorded and analyzed automatically using the ANY-MAZE software. Data were collected for 10 min.

### 5.8. Social Interaction Test

The apparatus consisted of a rectangular parallelepiped (30 × 60 × 40cm). Each mouse was placed in the box for 10 min to freely explore the area for habituation. In the sociability test, an unfamiliar C57BL/6N male mouse (stranger mouse) that had no previous contact with the subject mouse was put into one of the transparent cages (7.5 × 7.5 × 10cm, which had several holes with a diameter of 1 cm) located at the corners of each lateral compartment. The stranger mouse was enclosed in the transparent cage, which allowed nose contact through the bars but prevented fighting. The subject mouse was placed in the center to explore the entire box for a 6 min session (sociability test). One side of the rectangular area was identified as the stranger area and the other as the empty area. The amount of time spent in each chamber and around each cage during the 6 min sessions was measured. After the first 6 min session, a second unfamiliar mouse was placed in the chamber that had been empty during the first 6 min session. This second stranger was enclosed in an identical transparent cage. The subject mouse thus had a choice between the first, already-investigated mouse (familiar mouse), and the novel, unfamiliar mouse (stranger mouse). The amount of time spent in each chamber during the second 6 min session was measured as described above (social novelty preference test). Data were recorded on video and analyzed using the ANY-MAZE software.

### 5.9. Home-Cage Activity Test

For measurements of locomotion, the mice were acclimated to the single housing environment, and their behavior was monitored for 2 days. Locomotor activity data were measured using a photobeam activity system (ACTIMO-100; BRC Co., Nagoya, Aichi, Japan), and activity counts were recorded at 30 min intervals.

### 5.10. Cotton Bud Biting Test

Aggressive behavior was examined using the cotton bud biting test according to a previous study [51]. The mice were held in an experimenter's hand and a sterilized cotton bud was held close to their faces. Biting of the cotton bud was considered aggressive behavior. The mice were tested 10 times. Analysis was conducted on the total number of biting attacks.

### 5.11. Hot Plate Test

The hot plate test was used to evaluate nociception or sensitivity to a painful stimulus. Mice were placed on a hot plate at 55.0 ± 0.3°C, and the latency to the first hind-paw response was recorded. The hind-paw responses counted were foot shakes or paw licks.

### 5.12. Tail-Suspension Test

The tail-suspension test is an assay that is widely used in rodents to assess depression-like behavior [52]. Each mouse was suspended by the tail at 60 cm above the floor, in a white plastic chamber, using an adhesive tape placed <1 cm from the tip of the tail. Its behavior was recorded for 6 min. Images were captured via a video camera, and immobility time was measured. In this test, the “immobile period” was defined as the period when the animals stopped struggling for ≥1 s. Data acquisition and analysis were performed automatically using the ANY-MAZE software.

### 5.13. Porsolt Forced-Swim Test

The Porsolt forced-swim test is an assay that is widely used in rodents to assess depression-like behavior [53]. The apparatus for the Porsolt forced-swim test consisted of four Plexiglas cylinders (20cmheight × 10cmdiameter). The cylinders were filled with water (23°C) up to a height of 7.5 cm according to a previous study [53]. The mice were placed into the cylinders, and their behavior was recorded over a 6 min test period. Immobility time was evaluated using the ANY-MAZE software.

### 5.14. Statistical Analysis of the Behavioral Tests

Statistical analysis was conducted using the SPSS software (IBM Corp., Tokyo, Japan). The data were analyzed using two-way analysis of variance (ANOVA) followed by Tukey's test, and two-way repeated measures ANOVA followed by Fisher's LSD test, Student's *t*-test, or paired *t*-test. A *p* value of <0.05 was regarded as statistically significant. Data are shown as box plots.

## Data Availability

All relevant data are within the manuscript.

## Abbreviations

ANOVA: Analyses of variance
BRC: Bio Research Center
Fisher's LSD: Fisher's least significant difference
NIH: National Institute of health.
